# Strategic organizational support for stress and burnout: insights from rural teachers' stress profiles

**DOI:** 10.3389/fpsyg.2025.1580573

**Published:** 2025-09-10

**Authors:** Xi Fan, Jianwei Fang

**Affiliations:** ^1^Department of Applied Psychology, School of Health Management, Guangzhou Medical University, Guangzhou, Guangdong, China; ^2^Department of Public Administration, Guangdong Teachers College of Foreign Language and Arts, Guangzhou, Guangdong, China

**Keywords:** rural teachers, perceived stress, occupational burnout, organizational support, latent profile

## Abstract

**Introduction:**

This study investigated the profiles of perceived stress among rural teachers in China and examined their associations with occupational burnout and organizational support.

**Methods:**

A random online survey of 6,105 rural teachers was conducted, yielding 6,000 valid responses. Standardized scales were used to assess perceived stress, teacher burnout, and organizational support. Latent Profile Analysis was applied to identify distinct stress profiles.

**Results:**

Four stress profiles emerged: High-Stress, Challenge, Balance, and Relaxed. Teachers in the High-Stress and Challenge groups reported higher levels of burnout and lower organizational support, while those in the Balance and Relaxed groups reported lower stress and burnout and higher organizational support. Demographic factors such as gender, age, marital status, and education significantly influenced stress profile membership.

**Discussion:**

These findings highlight the heterogeneity of stress experiences among rural teachers and suggest the need for tailored interventions. Strategies such as workload reduction, psychological support, mindfulness training, mentoring, and enhanced organizational support are recommended to promote teacher well-being and support sustainable rural education.

## 1 Introduction

Rural teachers play a critical role in ensuring educational equity and quality, yet they often work under highly challenging conditions (Berry and Gravelle, 2013). Compared to their urban counterparts, rural teachers face not only tangible constraints such as limited educational resources, geographic isolation, and insufficient community support, but also heavier teaching loads, restricted professional development opportunities, and inadequate organizational support. These complex and intertwined contextual factors result in a diverse and multifaceted landscape of stress experiences among rural teachers, rather than a single, linear pattern. Systemic issues, including unequal resource allocation and social inequities, further enrich the types and mechanisms of stress experienced in rural contexts (Ogakwu et al., 2022). Previous studies have highlighted the paradox of teacher shortages in rural areas despite government policies aimed at recruitment and retention, underscoring the complex interplay of individual, systemic, and policy factors that exacerbate stress among rural teachers (Wang and Luo, 2024). High levels of perceived stress not only impact teachers' wellbeing but also negatively affect the quality of education they provide (Cavallari et al., 2024; Wettstein et al., 2021). Therefore, systematically identifying and understanding the typological characteristics of stress among rural teachers is crucial for developing effective support strategies, enhancing teacher wellbeing, and improving educational outcomes.

The present study employs Latent Profile Analysis (LPA) (Spurk et al., 2020), a statistical method designed to identify subgroups within a population based on shared characteristics. Recent studies have successfully used LPA to reveal distinct profiles of occupational stress and mental health in various contexts, such as employees (Gerber et al., 2014), nurses (Jenull and Wiedermann, 2015), and rural populations (Abbott et al., 2022). These applications demonstrate the utility of LPA for understanding population heterogeneity and guiding tailored support strategies. To explore the relationship between workplace stressors and teacher wellbeing, the study adopts the Job Demands-Resources model as a theoretical framework (Demerouti et al., 2001; Taris and Schaufeli, 2015; Huang et al., 2016). The Job Demands-Resources model posits that job demands (e.g., workload, emotional labor) contribute to stress and burnout, while job resources (e.g., organizational support, autonomy, professional development) buffer these negative effects and promote wellbeing. By combining LPA with the Job Demands-Resources model, this research examines the specific demands driving stress among rural teachers and identifies resources that can mitigate these effects.

The objectives of this study are to: (1) identify distinct perceived stress profiles among rural teachers using LPA; and (2) examine the relationships between these profiles, occupational burnout, and organizational support. Additionally, it investigates how demographic factors such as gender, marital status, professional title, age, education level, work experience, and income influence stress profile categorization. By offering a comprehensive analysis of rural teachers' stress profiles and their associated factors, this study contributes to the literature on teacher wellbeing and provides practical insights for targeted interventions. Such measures can enhance teacher retention, improve job satisfaction, and support the sustainable development of rural education.

## 2 The study

### 2.1 Participants

From January to March 2024, the Questionnaire Star online platform was used to distribute electronic questionnaires to 6,105 rural teachers through random sampling. Each participant could complete the questionnaire only once, and responses with incomplete data or duplicate entries (identified via IP address) were excluded from the analysis. In total, 6,000 valid questionnaires were obtained. According to data released by the Ministry of Education in 2018, the total number of rural teachers in China was approximately 2.9 million; thus, our sample accounts for about 0.2% of the national rural teacher population. All participants provided informed consent and were informed of the study purpose before completing the survey.

### 2.2 Methods

#### 2.2.1 General information questionnaire

A self-designed general information questionnaire was used to collect basic demographic and professional data, including gender, marital status, teaching level, professional title, age, education level, years of service, and salary.

#### 2.2.2 Perceived stress scale

The perceived stress scale (PSS), developed by Cohen and Williamson (1991) and translated into a Chinese version by Yang and Huang (2003), was used to measure stress levels. The scale includes 14 items covering two dimensions: tension and lack of control. Responses were scored on a 5-point Likert scale, with reverse scoring applied to items 4, 7, 9, 10, and 13. Higher total scores indicate higher levels of perceived stress, with scores above 25 indicating a health-risk stress state. The Cronbach's α coefficient for this scale in the study was 0.87.

#### 2.2.3 Primary and secondary school teacher burnout questionnaire

The Primary and Secondary School Teacher Burnout Questionnaire, developed by Xu (2003), was used to assess the degree of occupational burnout. The questionnaire comprises 15 items across three dimensions: emotional exhaustion, reduced accomplishment, and depersonalization, addressing teachers' emotional, achievement-related, and interpersonal burnout. It employs a 5-point Likert scale, with reverse scoring for items 3, 7, 8, 10, and 15. Higher total scores indicate more severe burnout. The Cronbach's α coefficient for this scale in the study was 0.89.

#### 2.2.4 Perceived organizational support questionnaire

The Perceived Organizational Support (POS) Questionnaire, adapted and developed by Chen and Chen (2008) based on the POS scale by Eisenberger et al. (1986), was used to evaluate organizational support. The scale includes 10 items covering two dimensions: emotional and instrumental organizational support. Responses were rated on a 7-point Likert scale, with higher scores indicating greater perceived organizational support. The Cronbach's α coefficient for this scale in the study was 0.97.

### 2.3 Statistical analysis

All statistical analyses were conducted using R 4.3.3. Latent profile analysis was performed with the Mclust and tidyLPA packages (Rosenberg et al., 2019), using maximum likelihood estimation and the bootstrap method to calculate confidence intervals.

The optimal number of profiles was determined iteratively, and model fit was evaluated using Bayesian Information Criterion (BIC), Akaike Information Criterion (AIC), Bayesian Likelihood Ratio Test (BLRT), and entropy. BIC and AIC are commonly used information criteria designed to balance model fit and complexity, with smaller values indicating better model fit. BLRT is a statistical test for comparing the goodness-of-fit between models with different numbers of profiles. It determines the optimal number of profiles by evaluating the log-likelihood difference between models with *k* and *k*−1 latent profiles. Entropy measures the classification accuracy of the model, ranging from 0 to 1, with values closer to 1 indicating better classification performance. Classification results were assessed for group differences using one-way ANOVA and Bonferroni tests, with a significance level of α = 0.05.

### 2.4 Common method bias test

This study collected data on all variables through self-reported questionnaires, which may introduce common method bias. To address this, Harman's single-factor test was used to evaluate common method bias (Zhonglin, 2020). In the factor analysis, all items measuring perceived stress, occupational burnout, and organizational support were included. The results identified six factors with eigenvalues >1, with the first factor accounting for 39.73% of the total variance, below the critical threshold of 40%. This indicates that common method bias is not a serious concern in this study.

## 3 Findings

### 3.1 Correlation analysis of occupational burnout, organizational support, and perceived stress

Pearson correlation analysis results are shown in Table 1. Perceived stress and its dimensions were significantly correlated with occupational burnout and organizational support. Perceived stress was positively correlated with occupational burnout (*r* = 0.68, *p* < 0.001), indicating that rural teachers experiencing higher levels of stress tend to report greater occupational burnout. Conversely, perceived stress was negatively correlated with organizational support (*r* = −0.44, *p* < 0.001), suggesting that teachers with higher levels of organizational support experience less stress. Additionally, organizational support was negatively correlated with occupational burnout (*r* = −0.49, *p* < 0.001), indicating that greater organizational support reduces occupational burnout among rural teachers.

**Table 1:** Means (standard errors) and correlations (*r*) of variables and their dimensions.

	**Mean (SE)**	**Tens**.	**LC**	**PS**	**EE**	**LA**	**Depers**.	**OB**	**ES**	**IS**	**OS**
**Tension (Tens.)**	**11.31 (4.70)**	**1.00**									
Lack of control (LC)	11.53 (5.60)	0.38^***^	**1.00**								
**Perceived stress (PS)**	22.84 (8.57)	0.80^***^	0.86^***^	**1.00**							
Emotional exhaustion (EE)	18.07 (5.66)	0.61^***^	0.51^***^	0.67^***^	**1.00**						
Low accomplishment (LA)	10.81 (2.94)	0.38^***^	0.51^***^	0.54^***^	0.53^***^	**1.00**					
Depersonalization (Depers.)	5.24 (1.91)	0.22^***^	0.28^***^	0.31^***^	0.47^***^	0.37^***^	**1.00**				
**Occupational burnout (OB)**	34.12 (8.73)	0.57^***^	0.56^***^	0.68^***^	0.93^***^	0.76^***^	0.65^***^	**1.00**			
Emotional support (ES)	33.49 (11.09)	−0.35^***^	−0.38^***^	−0.44^***^	−0.46^***^	−0.43^***^	−0.21^***^	−0.48^***^	**1.00**		
Instrumental support (IS)	15.20 (4.76)	−0.31^***^	−0.35^***^	−0.40^***^	−0.41^***^	−0.40^***^	−0.20^***^	−0.45^***^	0.87^***^	**1.00**	
**Organizational support (OS)**	48.69 (15.40)	−0.35^***^	−0.38^***^	−0.44^***^	−0.46^***^	−0.43^***^	−0.21^***^	−0.49^***^	0.99^***^	0.93^***^	**1.00**

Bold values indicate total scores of the corresponding constructs. ^*^p < 0.05, ^**^p < 0.01, ^***^p < 0.001.

### 3.2 Latent profile analysis of rural teachers' perceived stress

The analysis began with an evaluation of the feasibility of different variance-covariance structures using the mclust and tidyLPA packages. Four models were tested: EEI (Model 1), EEE (Model 3), VVI (Model 2), and VVV (Model 6) (see Wardenaar, 2021 for model descriptions). The results indicated that the EEI model demonstrated good convergence and classification performance (Bottomley et al., 2022; Howard, 2023). Analysis of EEI models with 1–5 profiles revealed that the 4-profile solution [BIC = 27,021.06, AIC = 26,933.97, BLRT (*p*) = 0.93, Entropy = 1.00] was optimal based on model fit indices (see Table 2). Under this classification, rural teachers surveyed had an 86% probability of belonging to one of the profiles, supporting the adoption of the 4-profile classification solution.

**Table 2:** Model fit indices for latent profile analysis with different numbers of profiles.

**Model**	**BIC**	**AIC**	**ILC**	**BLRT**	**BLRT (*p*)**	**Entropy**	**LogLik**
1	33,421.10	33,492.69	–33,447.90			1.00	–16,706.55
2	32,006.30	31,959.40	–33,972.43	1,467.64	0.01	0.60	–15,972.70
3	31,405.35	31,338.35	–33,863.67	627.33	0.01	0.65	–15,659.18
4	27,021.06	26,933.97	–27,021.08	–0.44	0.93	1.00	–13,453.98
5	27,049.07	26,941.88	–28,163.22	4,408.98	0.01	0.88	–13,454.94

**Figure 1:**
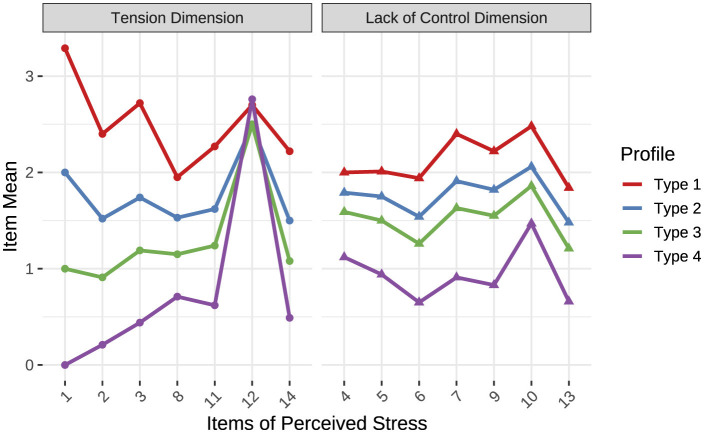
Latent profile analysis results of perceived stress among rural teachers. Higher scores on items 1, 2, 3, 8, 11, 12, and 14 indicate stronger tension, while higher scores on items 4, 5, 6, 7, 8, 9, 10, and 13 indicate stronger lack of control.

Based on the results of the latent profile analysis (see Figure 1), rural teachers' perceived stress was categorized into four latent types (1, 2, 3, 4).

#### 3.2.1 High-stress (type 1)

Teachers in this category scored the highest on tension and lack of control, indicating significant emotional stress. They may face work-related, familial, or other life challenges, requiring additional support and attention. The average perceived stress score for this group was 32.43, exceeding the health risk threshold of 25. This group was therefore named *High-Stress*. It included 1,008 individuals, accounting for 16.80% of the total sample.

#### 3.2.2 Challenge (type 2)

Teachers in this group also reported relatively high scores on tension and lack of control, reflecting considerable emotional stress. However, compared to the High-Stress group, these teachers may have slightly better coping abilities. With an average perceived stress score of 24.73, close to the health risk threshold, this group was named *Challenge*. It comprised 2,961 individuals, representing 36.60% of the sample.

#### 3.2.3 Balance (type 3)

Teachers in this category had lower scores on tension and lack of control, suggesting less emotional stress compared to the previous groups. This implies stronger psychological adaptability or a more stable and balanced state of life. The average perceived stress score for this group was 19.68, earning it the name *Balance*. A total of 2,161 individuals fell into this category, comprising 36.02% of the sample.

#### 3.2.4 Relaxed (type 4)

Similar to the Balance group, teachers in this category scored low on tension and lack of control, indicating minimal emotional stress. However, their average perceived stress score of 11.82 was notably lower than that of the Balance group, reflecting a more relaxed response to stress. Consequently, this group was named *Relaxed*. It included 635 individuals, accounting for 10.58% of the sample. Teachers in this category likely exhibit strong psychological adjustment abilities and a distinctly relaxed attitude toward stress.

### 3.3 Differences in perceived stress, occupational burnout, and organizational support across profiles

Significant differences were observed among rural teachers of different latent profiles in terms of mean scores for perceived stress, occupational burnout, and organizational support, including their respective dimensions (see Table 3). Teachers in the High-Stressprofile had the highest scores for perceived stress and occupational burnout, followed by the Challenge, Balance, and Relaxed profiles, with the Relaxed group scoring the lowest. *Post-hoc* tests confirmed that these differences were statistically significant (*p* < 0.05).

**Table 3:** Means and *post-hoc* test results of variables and their dimensions across different latent profiles of rural teachers' perceived stress.

**Variable**	**Profile 1**	**Profile 2**	**Profile 3**	**Profile 4**	***F***	***p***	**η^2^**	***Post-hoc* test**
Tension	17.54	12.39	9.09	5.23	2678.20	< 0.001^***^	0.57	1>2>3>4
Lack of control	14.88	12.34	10.60	6.59	381.10	< 0.001^***^	0.16	1>2>3>4
Perceived stress	32.43	24.73	19.68	11.82	1651.28	< 0.001^***^	0.45	1>2>3>4
Emotional exhaustion	23.51	18.88	16.32	12.62	836.39	< 0.001^***^	0.30	1>2>3>4
Low accomplishment	12.58	11.12	10.29	8.66	306.59	< 0.001^***^	0.13	1>2>3>4
Depersonalization	5.80	5.42	5.01	4.49	82.03	< 0.001^***^	0.04	1>2>3>4
Occupational burnout	41.88	35.42	31.62	25.76	729.78	< 0.001^***^	0.27	1>2>3>4
Emotional support	26.89	32.99	35.09	40.21	238.70	< 0.001^***^	0.11	4>3>2>1
Instrumental support	12.70	15.04	15.78	17.77	180.74	< 0.001^***^	0.08	4>3>2>1
Organizational support	39.59	48.03	50.86	57.98	234.67	< 0.001^***^	0.11	4>3>2>1

^*^p < 0.05, ^**^p < 0.01, ^***^p < 0.001.

Conversely, scores for organizational support, both total and dimensional, decreased progressively from the Relaxed to Balance, Challenge, and High-Stress profiles. These differences were also statistically significant (*p* < 0.05).

These findings indicate that teachers experiencing less stress receive higher levels of emotional and instrumental support, which helps them better handle work challenges and reduces occupational burnout. The significant role of organizational support in alleviating perceived stress and occupational burnout suggests that educational administrators should enhance emotional and instrumental support for teachers to improve their wellbeing and work efficiency.

### 3.4 Demographic characteristics across latent profiles of perceived stress

**Figure 2:**
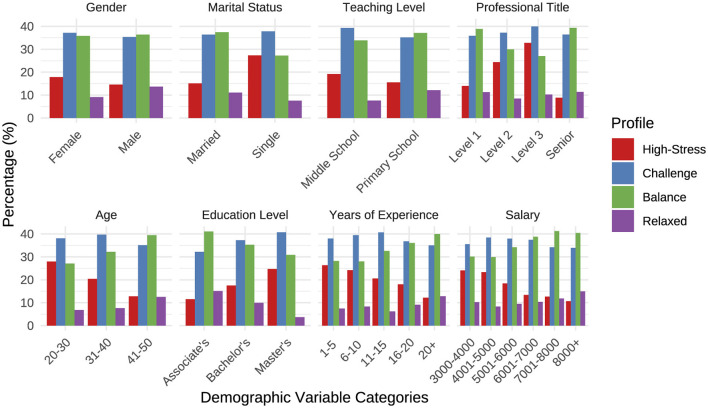
Demographic characteristics among rural teachers with different latent profiles of perceived stress.

**Figure 3:**
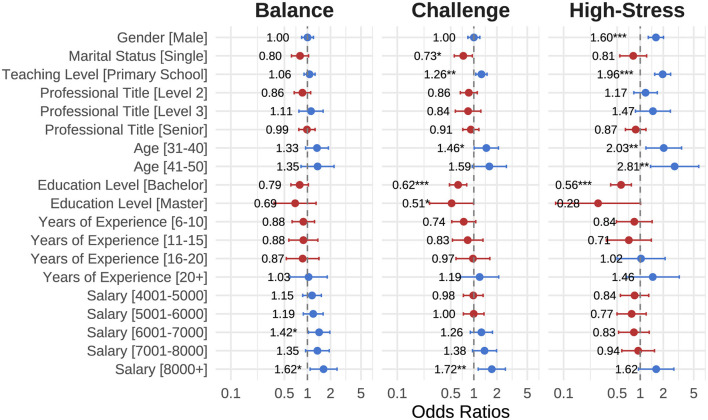
Odds ratios (95% confidence intervals) from multinomial logistic regression predicting stress profiles (reference group: Relaxed). Significant odds ratios (ORs) are indicated by asterisks (^*^p < 0.05, ^**^p < 0.01, ^***^p < 0.001).

The analysis initially revealed significant differences in demographic characteristics across the identified latent profiles of rural teachers' perceived stress (Figure 2). To clarify the complex relationships among these demographic factors and stress profiles, a multinomial logistic regression was performed with the “Relaxed” profile serving as the reference category (Figure 3).

Results showed that, compared to females, male teachers had significantly higher odds of belonging to the Relaxed type rather than High-Stress (*OR* = 1.60, *p* < 0.001). Unmarried teachers were less likely to be classified in the Challenge type (*OR* = 0.73, *p* < 0.05). Middle school teachers exhibited significantly higher probabilities of falling into Challenge (*OR* = 1.26, *p* < 0.01) and High-Stress profiles (*OR* = 1.96, *p* < 0.001), compared to primary school teachers. Regarding age, younger teachers had higher odds of being classified into higher stress profiles, notably teachers aged 31–40 years (*OR* = 1.46, *p* < 0.05) and those aged 41–50 years (*OR* = 2.03, *p* < 0.01). Teachers with higher educational attainment, particularly those with bachelor's (*OR* = 0.56, *p* < 0.001) or master's degrees (*OR* = 0.28, *p* = 0.052), were less likely to belong to the High-Stress type. In terms of income, teachers with salaries above 8000 RMB demonstrated significantly higher odds of being in the Balance (*OR* = 1.62, *p* < 0.05) and Challenge (*OR* = 1.72, *p* < 0.01) types compared to those with lower incomes.

Overall, these regression analyses confirm that demographic factors—including gender, marital status, teaching level, age, educational attainment, and salary—play distinct roles in determining teachers' stress profiles, providing targeted insights for intervention strategies.

## 4 Discussion

### 4.1 The impact of occupational burnout and organizational support on rural teacher stress profiles and targeted support strategies

The results indicate a significant positive correlation between perceived stress and occupational burnout among rural teachers, consistent with existing research from Spain, Sweden, and other countries (Portero de la Cruz et al., 2020; Gustafsson and Skoog, 2012). Prolonged exposure to high levels of stress has been identified as a major cause of mental health issues, such as anxiety and depression (Fedoce et al., 2018; Chourpiliadis et al., 2024). According to the Job Demands-Resources (JD-R) model, this high-stress state is primarily driven by job demands, including excessive workload and emotional labor, which are exacerbated by unique stressors in rural contexts, such as limited educational resources and inadequate social support. This underscores the need for systematic strategies to manage teacher stress, reduce the direct impact of job demands, and effectively prevent occupational burnout, alleviating its adverse effects, including fatigue, decreased job satisfaction, and potential teacher attrition.

The study identified a significant negative correlation between organizational support and both perceived stress and burnout, consistent with organizational support theory and with findings from international research in various sectors (Saadeh and Suifan, 2020; Ilyas et al., 2023). Teachers who receive higher levels of emotional support (e.g., care from colleagues and supervisors) and instrumental support (e.g., resources and training) report lower stress and greater wellbeing. This aligns with the JD-R model's proposition that job resources buffer the negative effects of job demands. The association between increased organizational support and reduced stress levels underscores the crucial role of job resources in mitigating the adverse impact of job demands and fostering teacher wellbeing. Therefore, educational administrators should prioritize strengthening organizational support systems and enhancing job resources, such as promoting autonomy and providing professional development opportunities, to systematically alleviate teacher stress and effectively prevent burnout.

The study further categorized rural teachers into four latent stress profiles: high-stress, challenge, balance, and relaxed. These profiles significantly differed in perceived stress, burnout, and organizational support levels. High-stress teachers reported the highest stress and burnout levels, coupled with the lowest organizational support, indicating an urgent need for targeted interventions to reduce stress. Challenge teachers, despite higher stress and burnout levels, demonstrated resilience, benefitting from organizational support to some extent but requiring close attention to prevent stress escalation. Balance and relaxed teachers exhibited lower stress and burnout levels alongside higher organizational support, reflecting effective stress management strategies and positive wellbeing. Sustained support and resource allocation are essential to maintain their stable and productive state.

Notably, the high consistency observed between different stress profiles and levels of burnout in our cross-sectional data provides a novel perspective on the relationship between stress and burnout. Although this study did not directly observe longitudinal transitions between stress profiles, existing theories and empirical research suggest that the relationship between stress and burnout is dynamic. For example, Dunford et al. (2012) found through longitudinal tracking that career transitions (such as promotion, job transfer, or onboarding) can lead to significant changes in burnout levels, with different burnout dimensions showing varying sensitivities to environmental changes. This indicates that teachers' stress and burnout states may undergo continuous transformations over the course of their careers, influenced by individual roles, organizational changes, and external circumstances. Drawing on the theoretical framework of the Job Demands-Resources (JD-R) model, which emphasizes the dynamic balance between job demands and resources, we propose that there is a realistic “fluid space” or “transitional state” between different stress types. This highlights the need for future longitudinal research to further elucidate the mechanisms underlying stress-to-burnout transitions and to optimize intervention strategies.

A key consideration is the directionality of the relationship between stress and organizational support: does lower stress enable teachers to seek and utilize more organizational support, or does greater organizational support directly reduce stress? While the cross-sectional design of this study cannot establish causality, it is plausible that teachers with lower stress are more likely to seek and benefit from support, which, in turn, further alleviates stress, creating a positive feedback loop. Future research should adopt longitudinal designs and combine experimental and field investigations to explore the dynamic mechanisms of how organizational support influences different stress profiles. This would uncover causal pathways and provide evidence for developing more effective targeted interventions.

To effectively address the diverse needs of rural teachers, targeted individual-level interventions and systemic reforms are necessary. For High-Stress teachers, immediate support should include reducing administrative tasks to optimize workload, providing psychological counseling and mindfulness training to improve stress management, and establishing peer support and mentorship programs to foster a supportive school environment. Challenge teachers, though resilient, require targeted professional training in areas such as behavior management and technology integration, coupled with opportunities for decision-making participation, leadership roles, and recognition to enhance motivation and professional fulfillment. Balance and Relaxed teachers, despite their relative stability, benefit from ongoing professional development opportunities, regular evaluations, and adjustments in support systems to sustain their positive states and prevent complacency. Additionally, promoting work-life balance and open communication can further enhance their wellbeing and job satisfaction. Systemic reforms, including increased educational funding, government-subsidized training programs, improved working conditions, upgraded infrastructure, and better housing and amenities, are essential for addressing broader stress factors and improving the overall work environment. Such comprehensive measures will significantly enhance rural teacher wellbeing, job satisfaction, and educational outcomes.

### 4.2 The impact of demographic variables on rural teacher stress profiles and targeted support strategies

Research indicates that demographic factors exert multi-layered and multidimensional influences on the formation of stress typologies among rural teachers. These variables not only reflect teachers' individual characteristics but also profoundly illustrate how sociocultural and organizational contexts shape stress experiences. As key external determinants, demographic features help elucidate intra-group differences among teachers and provide theoretical support for tailored interventions and policy-making.

The findings demonstrate that gender and marital status significantly influence stress typologies among rural teachers. This may be attributed to the fact that female teachers often face the dual pressures of professional and family responsibilities—pressures that are exacerbated in rural settings by limited resources and social support. Therefore, it is crucial to prioritize gender-sensitive and life-stage-appropriate support measures for such groups, including mentorship programs, flexible work arrangements, and psychosocial support networks. In addition, unmarried teachers were less likely to be classified as “challenge type,” suggesting that marital status not only affects the number of stressors but may also influence teachers' perceptions of and coping responses to stress. Unmarried teachers, who are often in the early stages of their careers, may face different role expectations or experience less family-related stress, resulting in distinct stress experiences compared to their married counterparts. These findings suggest that school administrators should carefully consider the unique pressures and needs associated with different marital and career stages to achieve more refined and effective intervention strategies.

Teaching level and age also play significant roles in shaping teachers' stress experiences. Secondary school teachers tend to experience higher occupational stress due to greater curricular complexity and increased student management responsibilities. Younger teachers, particularly those at the outset of their careers, are more susceptible to stress owing to immature coping mechanisms and limited support systems. Accordingly, specialized career development, targeted pre-service training, and resilience-building initiatives are essential for supporting teachers at different levels and stages.

The effect of income is more complex and diverse. Teachers with higher incomes are more likely to be classified as either “balanced type” or “challenge type.” This finding suggests that increased income does not function as a singular “protective factor” but may also bring new sources of stress and adaptive demands by affording greater professional engagement opportunities and achievement motivation. Thus, higher income reflects the diversification of stress experiences and adaptation strategies, rather than providing a uniform protective effect.

Importantly, our study found that professional title and years of service were not significant predictors of teacher stress typologies. In the rural education context, traditional markers of career advancement and tenure may not effectively alleviate occupational stress or enhance adaptive capacity. This may be related to the widespread challenges faced by rural teachers, such as limited external resources, constrained career development, and scarce promotion opportunities. Even with extensive teaching experience, teachers may not experience substantial relief from stress if they lack effective support systems. These results further underscore that contextual, organizational, and social factors—such as school climate, administrative support, and community involvement—may be more critical than individual career trajectories. In rural settings, seniority alone is insufficient to counteract structural stressors; supportive policies and environmental improvements are likely the key to enhancing teachers' stress resilience.

Taken together, these findings highlight the urgent need to move from a “homogenized” to a “precision-based” support system for rural teachers. Administrators and policymakers should fully consider teachers' diverse backgrounds and actual needs when designing support measures. Such targeted and context-sensitive strategies not only help alleviate occupational stress, prevent burnout, and enhance workforce stability and wellbeing, but also contribute to the high-quality development of rural education.

## 5 Conclusion

This study employed latent profile analysis to comprehensively reveal the diversity of perceived stress among Chinese rural teachers and its relationship with occupational burnout and organizational support. Rural teachers' perceived stress was categorized into four profiles: *High-Stress, Challenge, Balance*, and *Relaxed*. These classifications provide a critical basis for designing personalized support strategies tailored to different stress profiles. The findings indicate that perceived stress significantly impacts occupational burnout, while organizational support plays a key role in alleviating stress and reducing burnout. Significant demographic differences were observed across profiles, highlighting the necessity of individualized support policies. Educational administrators should enhance organizational support systems to help teachers manage stress and challenges, thereby improving their wellbeing and efficiency, and contributing to the sustainable development of educational quality and the broader education sector.

However, this study has certain limitations. The sample may not fully represent rural teachers nationwide. As with all online surveys, uneven internet access in some rural areas may have affected participation and sample representativeness. Additionally, this study did not consider individual characteristics such as personality traits, emotional intelligence, or coping styles, which may also influence stress and burnout. Future research should expand sample size and geographic coverage, incorporate a broader range of personal factors, and employ longitudinal designs to improve generalisability and deepen understanding.

## Data Availability

The original contributions presented in the study are included in the article/supplementary material, further inquiries can be directed to the corresponding authors.
